# Pathogenicity of Three Entomopathogenic Fungi to *Matsucoccus matsumurae*

**DOI:** 10.1371/journal.pone.0103350

**Published:** 2014-07-28

**Authors:** Weimin Liu, Yingping Xie, Jing Dong, Jiaoliang Xue, Yanfeng Zhang, Yaobin Lu, Jun Wu

**Affiliations:** 1 College of Life Science, Shanxi University, Taiyuan, China; 2 Institute of Plant Protection and Microbiology, Zhejiang Academy of Agricultural Sciences, Hangzhou, China; 3 Forest Pest Quarantine Station of Jinhua Municipal Forestry Bureau, Jinhua, China; Oregon State University, United States of America

## Abstract

*Matsucoccus matsumurae* (Kuwana) (Hemiptera: Coccoidea: Matsucoccidae) is an invasive alien species and a destructive pest of two native Chinese pines, *Pinus tabulaeformis* Carr. and *P. massoniana* Lamb., throughout the eastern regions of China. The pathogenicity of three entomopathogenic fungi, *Lecanicillium lecanii* strain V3.4504 and V3.4505, *Fusarium incarnatum-equiseti* strain HEB01 and *Lecanicillium fungicola* strain HEB02, against *M. matsumurae* was tested in four instars, to evaluate their potential as a biological control agent. The results showed that the four strains caused disease and death of the scale insect, among which the *L. lecanii* strains V3.4504 and V3.4505 displayed stronger virulence than the *F. incarnatum-equiseti* strains HEB01 and *L. fungicola* strain HEB02 to *M. matsumurae* in the 2^nd^-instar nymphs and the adult females. Furthermore, *L. lecanii* V3.4505 was most virulent to *M. matsumurae*. The adult females and the male 3^rd^-instar nymphs of *M. matsumurae* were susceptible to *L. lecanii* V3.4505; the adult females were more susceptible at LT_50_ = 1.96 than the 3^rd^-instar nymphs at LT_50_ = 5.67. The body surface structure, cuticle thickness and wax secretions of *M. matsumurae* impacted the fungal infection. *L. lecanii* is a promising biocontrol agent, and newly emerged male 3^rd^-instar nymphs and adult females are a crucial period of the insect’s life cycle for *M. matsumurae* biocontrol.

## Introduction

Pine bast scales are a group of destructive insect pests in pine forests in northern America, eastern Asia and Europe. Pine bast scales are classified as belonging to the family Matsucoccidae in the Coccoidea of Hemiptera. Matsucoccidae contains one extant genus, *Matsucoccus* Cockerell, in which 39 species have been recorded around the world. Japanese pine bast scale, *Matsucoccus matsumurae* (Kuwana), is the type species of this genus [1], [2]. *M. matsumurae* was first identified in 1905 by Kuwana in Tokyo, Japan, and its original host is the Japanese black pine, *Pinus thunbergii* Parlatore [3]. It first spread abroad from Japan and to the Korean Peninsula. In the mid-1940s, this species was discovered at Lushun in Liaoning Province of the northeastern China and at Yantai in Shandong Province. Prior to the 1970s, *M. matsumurae* was found throughout Liaoning Province in northeast China and spread to Shanghai, Jiangsu and Zhejiang in eastern China [4]. In these new territories, *M. matsumurae* adapted well to the two Chinese native pine species, *Pinus tabulaeformis* Carr. and *P. massoniana* Lamb., which allowed its population to rapidly expand and resulted in serious damage to the pine forests at a large scale. The damaged trees showed bark dehiscence, needle defoliation, twig wilt, treetop droop, and even death. Such heavy infestations attracted considerable attention from forest managers, scientists and governments. The State Forestry Department of China promulgated *M. matsumurae* to be a quarantine pest and the worst destructive exotic forest pest. Many chemical insecticides were used to control this pest, but their effectiveness was limited. By 1990, up to 333,000 hm^2^ of pine forests had been heavily damaged, and 133,000 hm^2^ of pine trees were cut down to control the spread of this scale insect [5]. Despite these control measures, *M. matsumurae* has continued to damage over 70,000 km^2^ of pine forest annually in recent years. This is because of their particularities in metamorphism, development, wax secretion and biology. *M. matsumurae* exhibits sexual dimorphism, and the male and female have different metamorphism. The male scales developed in a complete metamorphism, that is, from eggs, 1^st^-instar nymph, 2^nd^-instar nymph, 3rd-instar nymph, prepupa, and pupa, to adult males. While the females developed in an incomplete metamorphism, that is, from 1^st^-instar nymphs, 2^nd^-instar nymphs directly to the adult females. Most of their life they live concealed under the phloem or inside bark crevices. They secrete wax mainly at later stage of the 1^st^-instar, 2^nd^-instar nymph, 3^rd^-instar nymph, and adult female [6].

Because of the difficulty of controlling *M. matsumurae* and environmental pollution resulting from chemical insecticides, biological control has been proposed. However, using pathogens as biological agent to control *M. matsumurae* has not yet been reported. The entomopathogenic fungi parasitized on scale insects was first studied in the late 19^th^ century. In 1861, Nivter identified the fungus *Verticillium lecanii* (Zimmermann) Viégas (now named *Lecanicillium lecanii*) as being able to parasitize a soft scale insect, *Lecanii coffeae* Walker (now named *Saissetia coffeae*) in Ceylon. Since then, numerous species and strains of entomopathogenic fungi associated with scale insects have been identified. To date, approximately 140 species within 55 genera of fungal pathogens of scale insects have been recorded worldwide, and some of these fungi have been used as biological control agents [7].

In the present study, four strains of three species of entomogenous fungi, *Lecanicillium lecanii* strain V3.4504 and strain V3.4505, *Fusarium incarnatum-equiseti* strain HEB01, and *Lecanicillium fungicola* strain HEB02 isolated from the scale insects and brown rice planthopper [8]–[10] were used to infect *M. matsumurae* in different developmental stages. The objective was to test the pathogenicity of the four strains to *M. matsumurae* and to determine the stages of the life cycle susceptible to fungal invasion, so as to evaluate their potential as a biological control agent.

## Materials and Methods

### Ethics Statement

The collection of *M. matsumurae* was permitted by Forestry Disease and Pest Control Station of Jinhua City, Zhejiang Province, China.

### Entomopathogenic fungi and scale insects

#### Fungi

*Lecanicillium lecanii* strain V3.4504 and *L. lecanii* strain V3.4505 were purchased from China General Microbiological Culture Collection Center; strain V3.4504 was originally isolated from a brown planthopper, and strain V3.4505 was originally isolated from a species of scale insect [9]. *Fusarium incarnatum-equiseti* strain HEB01 and *Lecanicillium fungicola* strain HEB02 were originally isolated by us from the brown soft scale, *Coccus hesperidum* L [8]. The culture and preparation of conidial suspensions were described in our previous methods by Liu et al. [9]. The concentration of the conidial suspension used for infection was 5×10^7^ conidia/ml.

#### Scale insect

The Japanese pine bast scale *M. matsumurae* was collected from the forest of *Pinus massoniana* in Jinhua (N 28°32′, E 119°14′) of Zhejiang Province in China during late March to mid-April. During this time, *M. matsumurae* emerged from its overwintering, and the male scales developed from the 2^nd^-instar nymph stage, through male 3^rd^-instar nymph stage, male prepupa and pupa stage, to adult males, while the females developed from the 2^nd^-instar nymph stage directly to the adult female stage. Among these instars, the adult males were not chosen as their life span is only 1 or 2 days. The twigs containing the scale insects were cut in the forest, sorted by development stages and brought to the laboratory. Scale insect samples were then selected for the infection assays.

### Inoculation of *M. matsumurae* at different developmental stages

First, the 2^nd^-instar nymphs and the adult females were tested to compare the virulence of the four strains from the three species to *M. matsumurae*. The tests distinguished one highly virulent strain to *M. matsumurae*. Second, the highly virulent strain was employed to infect the four instars of the scale insect, the 2^nd^-instar nymphs, male 3^rd^-instar nymphs, prepupae and adult females, in order to identify the stage of *M. matsumurae* that was most susceptible to the fungal invasion.

#### Inoculation of the second-instar nymphs

Post-hibernation, the 2^nd^-instar nymphs settled under the phloem or began to feed, their bodies grew bigger and they emerged at the bark cracks. During the testing, the twigs containing the scale insects were cut into short sections of 2–3 cm in length, and each section contained 5–10 individuals. The bark was removed to expose the scale insects, and the twigs were then immersed in the conidial suspension for 10 seconds to inoculate the scale insects with the fungus. Following inoculation, the scale insects were air dried for approximately 5 minutes and transferred into Petri dishes (9 cm in diameter) containing moistened filter paper for moisturizing.

#### Inoculation of the male 3^rd^-instar nymphs

The 2^nd^-instar nymphs molted into the male 3^rd^-instar nymphs. The newly emerged 3^rd^-instar nymphs were inoculated by immersing them in the conidial suspension for 10 seconds, followed by air-drying for approximately 5 minutes. Subsequently, they were transferred into a 1.5 mL Eppendorf tube, which was perforated with 9 small holes (approximately 0.1 mm in diameter) to facilitate insect respiration and gas exchange, and each tube contained 10 insect individuals and a pine twig (approximately 2 cm in length and 0.5 cm in diameter) for the insects to crawl on and parasitize. These tubes were transferred into Petri dishes (9 cm in diameter) containing moistened filter paper for moisturizing.

#### Inoculation of the male prepupae

The male 3^rd^-instar nymphs can secret wax filaments on their body surface to form a wax cocoon. In the wax cocoon, the scale insect enters the prepupae and pupae stages. The wax cocoons clustered under the bark cracks. The wax cocoons containing the prepupae were inoculated. When inoculating, the bark cracks were raised to expose the wax cocoons. The bark containing the prepupae was immersed in the conidial suspension for 10 seconds, and each section of bark contained 8–10 prepupae. Following inoculation, the bark and scale insects were air dried for approximately 5 minutes and transferred into Petri dishes containing moistened filter paper for moisturizing.

#### Inoculation of the adult females

The newly emerged adult females without wax filaments secreted on their surface were used for inoculation. The inoculation method of the adult females was the same as that of the male 3^rd^-instar nymphs.

Each test described above was repeated thrice, and each replication tested 50 insect individuals. The control tests for each instar were only treated with a sterile solution of 0.1% Tween-80. All of the Petri dishes containing inoculated samples were placed in a constant temperature and illumination incubator at 25±0.5°C, 75±10% RH, and a photoperiod of 14∶10 (L:D).

### Observation of the infective symptoms and count of the mortality of *M. matsumurae*

The infective symptoms of *M. matsumurae* at different developmental stages after inoculation were observed daily under a stereomicroscope, and photographs were taken using an Olympus C5050Z digital camera (OLYMPUS OPTICAL Co. Ltd). At the same time, the mortalities of each test group were counted. To compare the virulence of the four strains, the 2^nd^-instar nymphs were observed for 14 days, and the adult females were observed for 8 days. To compare the susceptibility of *M. matsumurae* in the four instars, the observations persisted for 8 days.

### Observation of the cuticle structure of *M. matsumurae* in different developmental stages

To study the effect of the insect surface structure and cuticular thickness on the fungal invasion, the cuticles of *M. matsumurae* in the four developmental stages were observed using scanning electron microscopy (SEM) and transmission electron microscopy (TEM). Insect samples were fixed in glutaraldehyde (2.5%, (V/V), prepared in 0.2 M phosphate buffer at pH 7.2) at 4°C for 48 h, and they were then prepared for SEM and TEM observation. 10 insect individuals were used for each observation. The detailed methods are described in Liu et al [9].

### Statistical analysis

The mortality of *M. mausumurae* in treated groups was corrected by that observed in the control groups. The median lethal time (LT_50_) was estimated using the probit analysis (SPSS 16.0). The means of the percent mortality were compared by Tukey-HSD test (P<0.05) using the ANOVA procedure of SPSS. Comparisons were made among the percent mortality of the same life stage infected by the four different strains and among the percent mortality of the four life stages infected by *L. lecanii* strain 3.4505, respectively.

Correlation analysis was used to statistically determine whether the cuticle thickness and wax layer (+/−) were related with LT_50_ and percent mortality. Correlated analysis was calculated by standard means of the cuticle thickness and the percent mortality, and the insect instars with or without wax layers were set to 1 and 0, respectively.

## Results

### Infective symptom of *M. matsumurae*

The symptoms of *M. matsumurae* in response to infection with the entomopathogenic fungus *Lecanicillium lecanii* strain V3.4505 were observed during the different instar stages.

**Figure 1:**
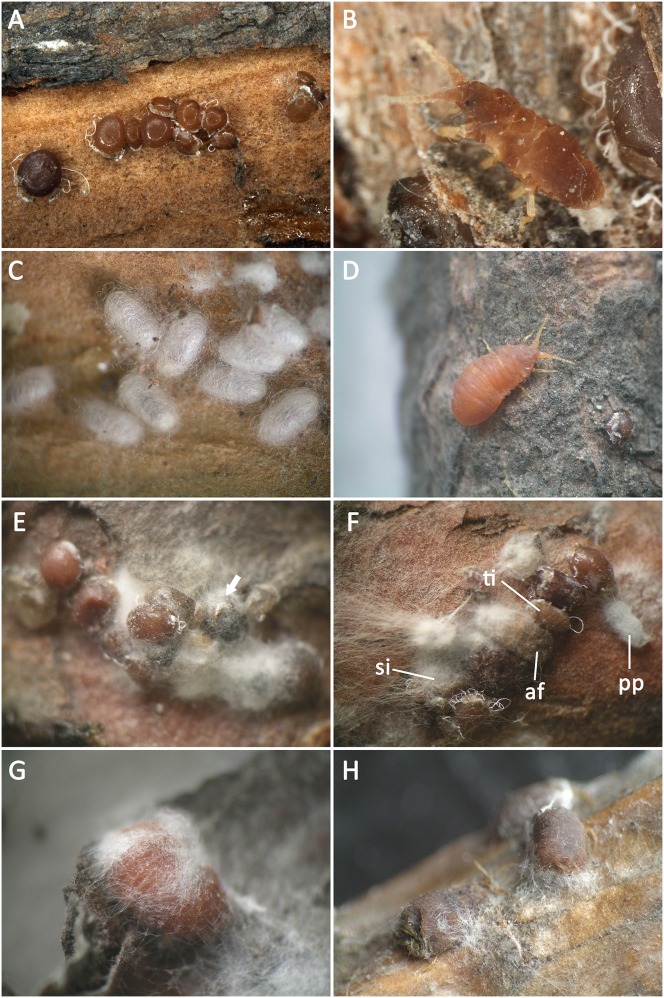
*M. matsumurae* and its infective symptom after being inoculated with the entomopathogenic fungus. A, the 2^nd^-instar nymphs settled down in clusters under the phloem; B, a newly emerged male 3^rd^-instar nymph; C, a cluster of the wax cocoons in which prepupae were concealed; D, newly emerged adult female; E, the infective symptom of the 2^nd^-instar nymphs, showing the mycelia covered the insect body, and the insect body color darkened (arrow); F, the second-instar nymphs (si) were inoculated, but the infective symptom were observed not only in this instar but also in the following instars, including the adult female (af), the male 3^rd^-instar nymph (ti) and the prepupae (pp) in the wax cocoon. The infected insects were easily distinguished from the population by mycelia; G, the adult female at the early stage of infection, showing the insect body did not obviously change despite the presence of some mycelia; H, the infective symptom of the adult females at the later stage of infection, showing their body color darkened.

Infective symptoms of the 2^nd^-instar nymphs: The 2^nd^-instar nymphs showed a pearl-shaped body without antennae and legs. They lived in clusters and were immobile (Fig. 1A). After inoculation, the nymphs showed two types of infective symptoms: 1) some mycelia appeared on the surfaces of nymphs within 24 h after inoculation. The mycelia gradually increased and covered the entire body of the insect, concentrating around the margin of the venter. As the mycelia spread, the neighboring individuals were invaded. At the later stage of infection, the diseased insect died, as indicated by the darkening of their body color (Fig. 1E). 2) The nymphs could molt and develop into the next instar. However, some individuals in the new instar stage showed disease symptoms, with mycelia emerging on the body surface (Fig. 1F).

Infective symptoms of the male 3^rd^-instar nymphs: The male 3^rd^-instar nymphs had developed antennae and thoracic legs and crawled actively (Fig. 1B). After inoculation, the 3^rd^-instar nymphs secreted wax filaments to form a wax cocoon in a short time before showing infective symptoms. However, despite being covered by the wax cocoon, the infected nymphs displayed symptoms, with a layer of mycelia appearing first on the insect body and then on the wax cocoon.

Infective symptoms of the prepupae: The prepupae were wrapped inside the wax cocoon (Fig. 1C). After inoculation, the diseased prepupae were observed to have a layer of mycelia extending over the cocoon.

Infective symptom of the adult females: Similar to the 3^rd^-instar nymphs in morphology, the adult females had also developed antennae and thoracic legs and crawled actively (Fig. 1D). After inoculation, the insects quickly showed infective characteristics. First, the vitality of the adult females declined rapidly, and a thin layer of mycelia was visible (Fig. 1G). As the mycelia increased, the diseased insects started to die and their body color darkened. As the mycelia propagated, the nutrition of the insects was exhausted, and the entire body of the insect shriveled (Fig. 1H).

### Comparison of the virulence of the four fungal strains infecting *M. matsumurae*

**Figure 2:**
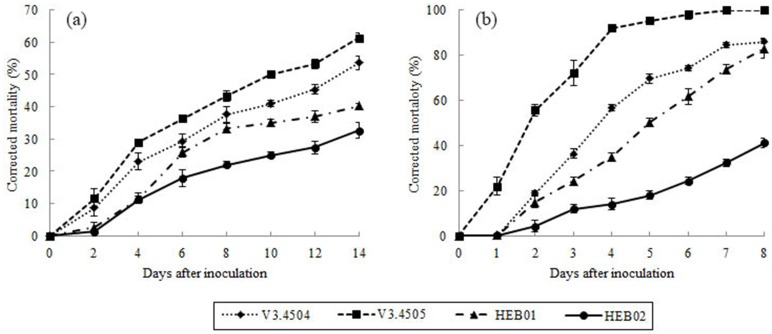
Corrected mortalities of *M. matsumurae* after inoculated with *L. lecanii*, *F. incarnatum-equiseti* and *L. fungicola*. A, 2^nd^-instar nymphs; B, adult females.

The 2^nd^-instar nymphs and adult females were tested in order to compare the virulence of the four strains to *M. matsumurae*. The results showed that all of the four strains were pathogenic to *M. matsumurae*, but their virulence differed (Fig. 2). The virulence of the fungus *L. lecanii* was higher than that of *F. incarnatum-equiseti* and *L. fungicola*. *L. lecanii* strain V3.4505 was the most virulent of the four strains from the three species, and its infection resulted in 61.33% mortality of 2^nd^-instar nymphs and 100% mortality of the adult females of *M. matsumurae*. Furthermore, the differences between strains were statistically significant (Table 1). Moreover, a probit analysis of the time mortality response revealed that the LT_50_ was 10.49 d for the 2^nd^-instar nymphs and 2.01 d for the adult females infected with *L. lecanii* strain V3.4505. In comparison, the LT_50_ values were 12.48 d and 4.24 d for the 2^nd^-instar nymphs and the adult females infected with *L. lecanii* strain V3.4504, respectively (Table 2). This finding indicated that strain V3.4505 could kill the 2^nd^-instar nymphs and the adult females more rapidly than strain V3.4504. The mortalities of the 2^nd^-instar nymphs in the other two test groups were less than 50% 14 d after inoculation with *F. incarnatum-equiseti* strain HEB01 or *L. fungicola* strain HEB02. However, *F. incarnatum-equiseti* strain HEB01 caused a mortality of 83.00% in adult females 8 d after inoculation, which was similar to the mortality caused by *L. lecanii* V3.4504. *L. fungicola* strain HEB02 only caused a mortality of 32.67% in the adult female (Table 1). Thus, *L. lecanii* V3.4505 was identified as a highly virulent strain against the 2^nd^-instar nymphs and the adult females of *M. matsumurae*.

**Table 1:** Average mortality of 2^nd^-instar nymphs and adult females after inoculated with the four strains.

Strain	2^nd^-instar nymph Mortality (%±SE)	Adult female Mortality (%±SE)
V3.4504	53.67±2.08c	86±1b
V3.4505	61.33±1.53d	100c
HEB01	40.33±0.58b	83±4.36b
HEB02	32.67±2.52a	41.33±2.08a

Note: Means followed by different letters in each column represents differ significantly between different strains (Tukey-HSD test: P<0.05).

**Table 2:** LT_50_ of the 2^nd^-instar nymph and the adult female of *M. matsumurae* after inoculated with the four strains.

Strain	Insect stage	LT_50_ (d)	95% Confidence limits	Regression equation
V3.4504	2^nd^-instar	12.48	12.73–13.11	Y = −1.236+0.099x
	Adult female	4.24	3.93–4.54	Y = −1.644+0.388x
V3.4505	2^nd^-instar	10.49	9.91–11.16	Y = −1.081+0.103x
	Adult female	2.01	1.81–2.18	Y = −1.218+0.607x
HEB01	2^nd^-instar	14.63	13.11–17.03	Y = −1.504+0.103x
	Adult female	5.17	4.98–5.38	Y = −1.915+0.37x
HEB02	2^nd^-instar	17.79	15.92–20.71	Y = −1.661+0.093x
	Adult female	8.79	8.34–9.34	Y = −2.134+0.243x

### Susceptibility of *M. matsumurae* in different developmental stages to entomopathogenic fungus

**Figure 3:**
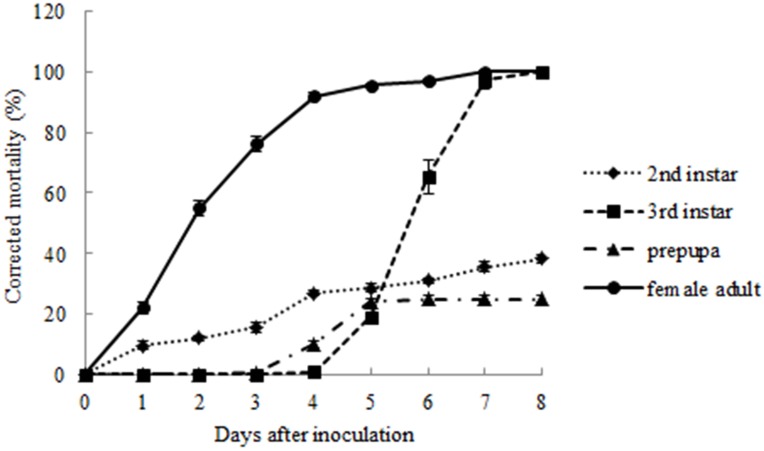
Corrected mortality of four developmental stages of *M. matsumurae* after inoculated with *L. lecanii* V3.4505.

The susceptibility of *M. matsumurae* at the four different instars to the entomopathogenic fungus was tested by inoculating them with *L. lecanii* V3.4505. The result showed that the susceptibility of *M. matsumurae* to *L. lecanii* V3.4505 varied with the insect developmental stage (Fig. 3). The 2^nd^-instar nymphs were less susceptible to fungal invasion, and their mortality was just 38.33% 8 days after inoculation; the prepupae were least susceptible to fungal invasion, and their mortality was only 25%; the male 3^rd^-instar nymphs and adult females were most susceptible to the fungal infection, and their mortalities all reached 100% 8 days after inoculation (Table 3). Comparatively, the LT_50_ of adult females was 1.96 d, which was shorter than the LT_50_ (5.67 d) of male 3^rd^-instar nymphs. This difference indicated that the adult females died more rapidly after being infected with *L. lecanii* V3.4505 than the male 3^rd^-instar nymphs; thus, the adult female stage of *M. matsumurae* was more susceptible to the fungal infection.

**Table 3:** Mortality and LT_50_ of *M. matsumurae* at different developmental stages inoculated with *L. lecanii* V3.4505.

Insect stage	Mortality (%±SE)	LT_50_ (days)	95% Confidence limits	Regression equation
2^nd^-instar	38.33±1.53b	9.63	8.76–10.92	Y = −1.331+0.138x
Male 3^rd^-instar	100c	5.67	5.69–5.74	Y = −7.842+1.383x
Prepupa	25±1a	9.64	8.53–11.76	Y = −2.344+0.243x
Adult female	100c	1.96	1.76–2.15	Y = −1.174+0.598x

Note: Means followed by different letters in a column represents differ significantly between each life stage (Tukey-HSD test: P<0.05).

### Effect of the cuticle of *M. matsumurae* on fungal invasion

**Figure 4:**
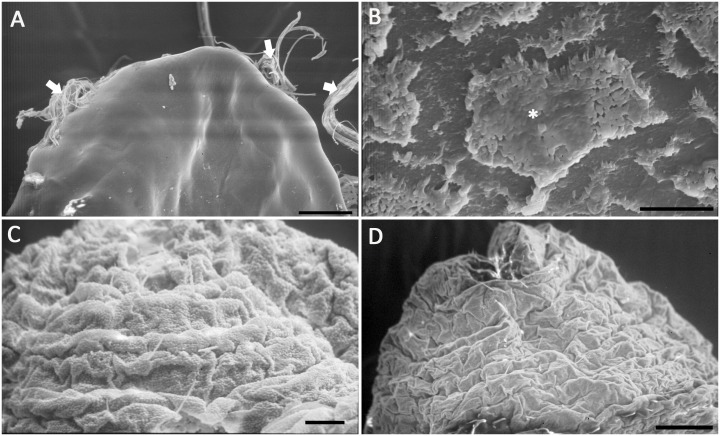
Scanning electron micrographs of the surface morphology of *M. matsumurae* at different developmental stages. A, 2^nd^-instar nymph, showing the insect body was smooth with wax filaments secreted from spiracles on both sides. Scale bar = 100 µm; B, magnified view of the 2^nd^-instar nymph, showing the thin wax layer on the insect surface. Scale bar = 10 µm; C and D, male 3^rd^-instar nymph and adult female, showing both of their body segments are distinct with obvious intersegmental folds. C, Scale bar = 20 µm. D, Scale bar = 100 µm.

The surface structure of *M. matsumurae* was observed using SEM technology. The results revealed that the body surface varied at different developmental stages. The surface of the 2^nd^-instar nymphs was smooth at 180× (Fig. 4A), while a thin wax layer on the insect surface was visible when magnified to 2500× (Fig. 4B). Both the male 3^rd^-instar nymphs (Fig. 4C) and adult females (Fig. 4D) showed distinct body segments and obvious intersegmental folds.

**Figure 5:**
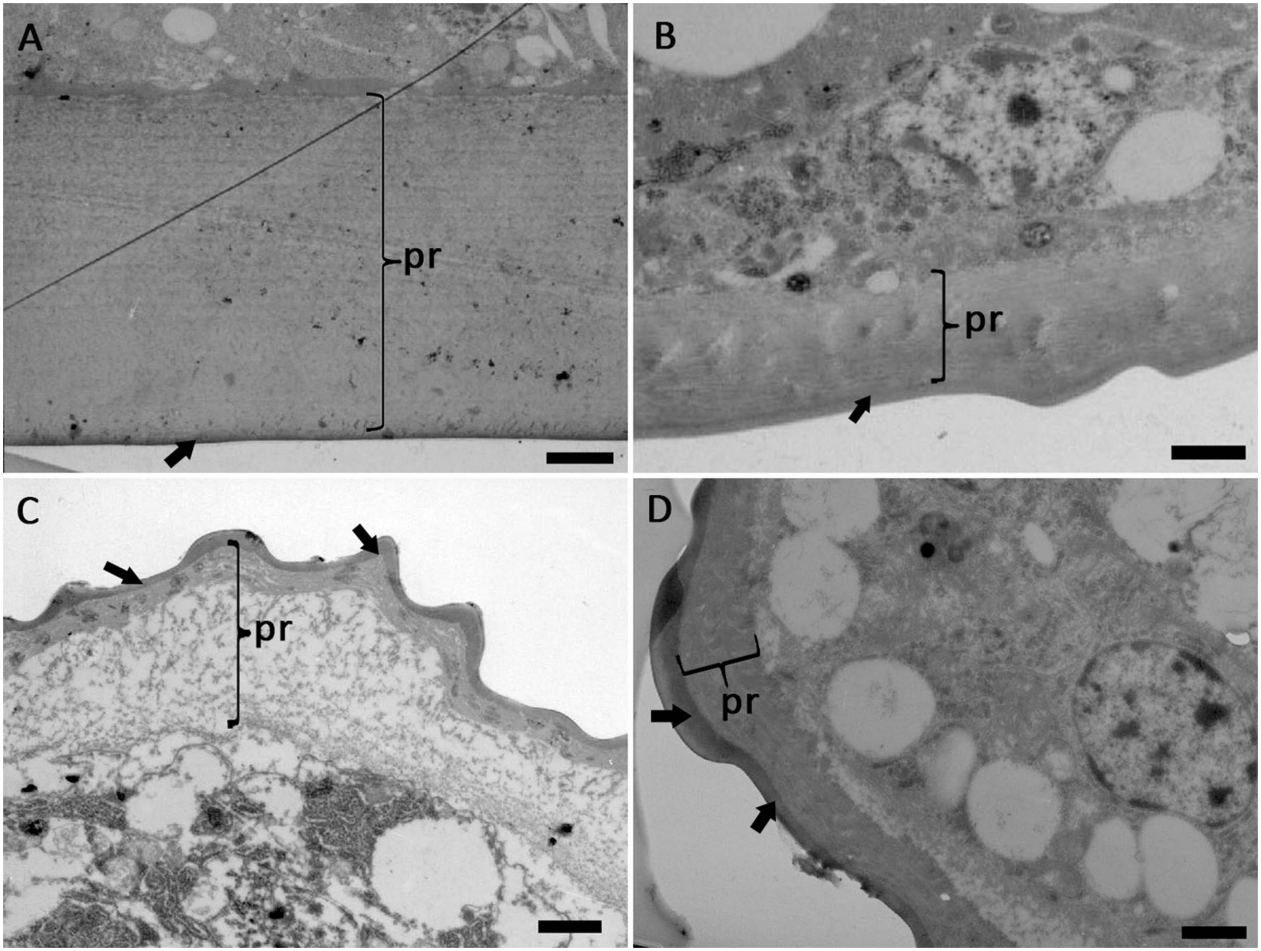
TEM photograph of the integument of *M. matsumurae*. (A) 2^nd^-instar nymph, scale bar = 2 µm; (B) male 3^rd^-instar nymph, scale bar = 1 µm; (C) prepuae, scale bar = 1 µm; (D) adult female. scale bar = 1 µm. Note: arrow points to the epicuticle, pr- procuticle.

The cuticle layers of *M. matsumurae* at different developmental stages were observed via TEM. The results showed that the cuticle of *M. matsumurae* consisted of a thin epicuticle and a very thick procuticle (Fig. 5), and the cuticular thickness, especially the thickness of the procuticle, varied greatly with the insect development stage (Table 4). As surrounded by the wax cocoon, the prepupa was counted out when using correlation analysis. Statistical analysis showed that the cuticle thickness had linear negative correlation with the percent mortality (r = −0.999, P<0.05), and had positive correlation with LT_50_ (r = 0.855, P = 0.345>0.05), but there was no linear correlation between the cuticle and LT_50_.

**Table 4:** The cuticle thickness (µm) of *M. matsumurae* in different developmental stages.

Cuticle layer	2^nd^-instar nymph	Male 3^rd^-instar nymph	Prepupa	Adult female
Cuticle	10.61–12.53	0.80–1.42	2.01–2.94	0.81–2.89
Epicuticle	0.11–0.38	0.11–0.38	0.10–0.33	0.11–0.68
Procuticle	10.52–12.21	0.69–1.11	1.81–2.63	0.42–2.37
Mortality (%)	38.33±1.53	100.00	25.00±1.00	100.00

The prepupa constitutes an exceptional development stage; its cuticular thickness was much thinner, but the mortality at this stage was only 25%. This low rate may be related to the wax cocoon that covered the prepupae and obstructed the fungal invasion. Correlation analysis showed that the wax layer (+/−) had linear negative correlation with the percent mortality (r = −0.991, P<0.01), and had positive correlation with LT_50_, (r = 0.912, P = 0.088>0.05), but there was no linear correlation between the wax layer and LT_50_.

## Discussion

In the present study, the pathogenicity of the entomopathogenic fungi *Lecanicillium lecanii* strains V3.4505 and V3.4504, *Fusarium incarnatum-equiseti* strains HEB01, and *L. fungicola* HEB02 to *M. matsumurae* was tested at four developmental stages. The results revealed that the four strains of the two fungi could infect and cause disease and death in *M. matsumurae*, but their virulence differed. The virulence of the fungus *L*. *lecanii* was higher than that of *F. incarnatum-equiseti* and *L. fungicola*. The pathogenicity of strain V3.4505 of *L*. *lecanii* against *M. matsumurae* (61.33% in mortality) was higher than that of strain V3.4504. Moreover, the fungus *F. incarnatum-equiseti* strain HEB01 was more pathogenic to *M. matsumurae* (40.33% in mortality) than *L. fungicola* strain HEB02. Thus, *L. lecanii* is considered a promising fungus to serve as a biocontrol agent against *M. matsumurae*, and the *L. lecanii* strain V3.4505 may be the better choice for this purpose; it not only resulted in higher mortality rates in the insect, but it also caused insect death more quickly.

The susceptibility of *M. matsumurae* to the entomopathogenic fungus was determined by infecting the insects with *L. lecanii* strain V3.4505. Of the four development stages, the 2^nd^-instar nymphs were less susceptible to the fungi, with a lower mortality of 38.33%. The prepupa stage was least susceptible, with the lowest mortality of 25%. The male 3^rd^-instar nymphs and the adult females were the most susceptible instars, both of their mortalities reached 100% after inoculation with *L. lecanii* strain V3.4505. However, the adult females died more quickly after inoculation. This phenomenon may be understood by comparing the difference in the cuticle structure of the scale insect.

Entomopathogenic fungi are known to most commonly invade their host through the external integument, including conidia attaching to the cuticle, germinating and hyphae penetrating the cuticle [11]. Pedrini et al. noted that surface structure and the chemical composition of the host cuticle affect the attachment of fungal propagules to the cuticle [12]. In the present infection experiment of *M. matsumurae*, the 2^nd^-instar nymphs were pearl-shaped and showed a thin wax layer on their body surfaces, and the prepupae were concealed inside the wax cocoon. Some studies have examined the wax substances of the scale insects. Wax substances usually consist of multiple components, including long-chain alkanes, fatty acids, fatty alcohols, esters, cyclic alcohols and cyclic acids, etc. These diverse compounds serve to protect the insect by inhibiting entomopathgenic invasion [13], [14]. In our previous studies on the histopathological changes of the two soft scales *Ceroplastes japonicus* Green and *Coccus hesperidum* L., the wax test or wax covering of scale insects played a function in inhibiting fungal infection [9], [10]. Mauchline, et al., who studied the process of infection of latania scale insect by *Cosmospora* sp., suggested that the wax test provided a physical barrier to fungal entry [15]. James et al. found that cuticular lipids of silverleaf whitefly affect conidial germination of *Beauveria bassiana* and *Paecilomyces fumosoroseus* [16]. In the present study, correlation analysis showed that the wax layer has linear negative correlation with the percent mortality of *M. matsumurae*. The wax layer covering on the 2^nd^-instar nymph surface and the wax cocoon surrounding the prepupae may have contributed to encumber the fungal attachment, germination and invasion, and that resulted in significantly lower mortality rates in these two instars. In contrast, the newly molted 3^rd^-instar nymphs and the newly emerged adult females had a higher mortality. It may be due to both instars without wax substance covering.

Boucias and Pendland suggested that the susceptibility of different larval instars to entomopathogenic fungi can be explained by their cuticular characteristics [17]. In our study, correlation analysis proved a linear negative correlation between the cuticle thickness and the percent mortality of *M. matsumurae*. The 2^nd^-instar nymphs of *M. matsumurae* have thicker cuticle, and their mortality was lower, while, the male 3^rd^-instar nymphs and the adult females have thinner cuticle, and their mortality was higher. It indicated that the thicker cuticles may result in fungal penetration through the cuticle more difficult. The insect cuticle, especially the procuticle, is a polymer network composed of chitin (a polysaccharide) embedded in a protein matrix. The cuticle must be breached by the mechanical pressure of hyphae and the action of cuticle-degrading enzymes produced by the fungus [11]. Thus, the thicker cuticles with more protein and chitin may make the fungus secret more extracellular enzymes and consume much longer time to degrade the cuticle. So, the 2^nd^-instar nymph stage showed a longer LT_50_ (9.63). Similarly, the male 3^rd^-instar nymphs and the adult females of *M. matsumurae* have higher mortality and shorter LT_50_ may be associated with their relatively thinner cuticle layer. However, differences in the ability to break down the cuticle between different species or strains of the fungi need further study by measuring the activities of specific enzymes produced by the fungi.

Besides with thinner cuticle, the male 3^rd^-instar nymphs and the adult females of *M. matsumurae* possessed distinct body segments and intersegmental folds, which favored the attachment of the fungal conidium. Furthermore, their intersegmental membranes were thinner, which allowed the fungus to penetrate more easily through the integument and enter the hemocoele. Butt et al., and Hajek and Eastburn noted that the adhesion of conidia to the cuticle can be region-specific, e.g. to intersegmental membranes [18], [19]. McCauley et al. found higher infection rates through intersegmental folds and ascribed this phenomenon to the protection of conidia by intersegmental folds, which provided a microclimate with a higher humidity to enhance conidia germination [20]. Pilz et al. studied the western corn rootworm *Diabrotica virgifera virgifera* Leconte and noted that sites with thinner cuticles, such as the intersegmental membranes, are more suitable for fungal penetration than other places [21]. These discoveries are similar to our results on the scale insect.

In summary, this study is the first report of the pathogenicity of entomopathogenic fungi on *M. matsumurae*. In the four tested strains, *L. lecanii*, strain V3.4505 was most pathogenic to *M. matsumurae*. The newly emerged male 3^rd^-instar nymphs and the adult females are the two susceptible stages to fungal invasion. The cuticle thickness and wax secretions of *M. matsumurae* are two primary factors impacting fungal infection. According to our observation, the life cycle and developmental schedule of *M. matsumurae* differ by the climatic region. In southern China, represented by Jinhua of Zhejiang Province, biological control of *M. matsumurae* should be conducted during late March and early April. In the northeast, such as Liaoning and Jilin Provinces, and the east, such as Shandong Province, the suitable control period should be delayed approximately one and a half months.
